# Disrupting *Salmonella* colonization and resistance: probiotic cell-free supernatants inhibit biofilm formation and invasion in meat and human isolates

**DOI:** 10.3389/fcimb.2026.1889179

**Published:** 2026-10-02

**Authors:** Azza S. El-Demerdash, Mervat A. Said, Mahmoud M. Hazaa, Mahmoud M. Amer, Yasmen F. Mahran, Hadil Alahdal, Munirah M. Alhammadi, Ahmed G. Abdelhamid

**Affiliations:** 1 Biotechnology Department, Animal Health Research Institute (AHRI), Agricultural Research Center (ARC), Zagazig, Egypt; 2 Botany and Microbiology Department, Faculty of Science, Benha University, Benha, Egypt; 3 Research Department, Natural and Health Sciences Research Center, Princess Nourah bint Abdulrahman University, Riyadh, Saudi Arabia; 4 Department of Biology, College of Science, Princess Nourah Bint Abdulrahman University, Riyadh, Saudi Arabia

**Keywords:** biofilm inhibition, invasion attenuation, one health, organic acid synergy, pan-drug resistance, postbiotics, Salmonella enterica, type III secretion system (T3SS)

## Abstract

**Background:**

*Salmonella enterica* remains a paramount global public health threat due to the rapid emergence of multi-drug and pan-drug resistant (PDR) strains transmitted through the food supply chain. This study adopted a One Health cross-sectional framework to investigate the occurrence and biocontrol of PDR *Salmonella enterica* serovars (*S.* Enteritidis, *S.* Kentucky, and *S.* Anatum) isolated from clinical gastroenteritis cases and retail meat products in Egypt.

**Methods:**

Clinical samples (n=20) from gastroenteritis patients were screened for PDR *Salmonella* and confirmed via *invA* gene PCR in addition to six PDR isolates previously from meat samples. Biocontrol efficacy was evaluated using cell-free spent media (CFSM; postbiotic preparations) derived from a consortium of probiotic strains. Chemical profiling of CFSM was executed via High-Performance Liquid Chromatography (HPLC). Antimicrobial activity, minimum inhibitory concentrations, and biofilm formation were quantified. Host cell invasion was assessed using a gentamicin protection assay in human Caco-2 intestinal epithelial cells. Ultrastructural alterations were examined via Transmission Electron Microscopy (TEM). Transcriptomic mechanisms were elucidated via RT-qPCR targeting biofilm regulators (*csgD*, *sdiA*) and *Salmonella* Pathogenicity Island 1 (SPI-1) invasion genes (*hilA*, *invF*, *prgH*, and *sipA*).

**Results:**

CFSM from *Lactobacillus helveticus* EMCC 1654 and *Lactobacillus plantarum* CCUG 30503 demonstrated marked antibacterial and antibiofilm activity. At sub-inhibitory concentrations (10% v/v), CFSM significantly attenuated biofilm formation and reduced host-cell invasion efficiency in *S.* Kentucky to 0.08% relative to untreated controls. TEM micrographs confirmed severe cell envelope disintegration, membrane detachment, and cytoplasmic leakage in CFSM-treated cells. RT-qPCR analysis revealed significant downregulation of the master biofilm regulator *csgD* (fold-change: 0.002) and the quorum-sensing receptor *sdiA* (fold-change: 0.02). Furthermore, key SPI-1 invasion genes were markedly repressed, including *hilA* (0.188), *invF* (0.001), *prgH*, and *sipA* (0.037). Quantitative HPLC profiling of *L. plantarum* CCUG 30503 CFSM- the lead candidate for downstream mechanistic analysis- identified a multi-organic acid cocktail dominated by lactic (1.89 mg/mL), succinic (1.53 mg/mL), and acetic (1.11 mg/mL) acids.

**Conclusion:**

*Lactobacillus*-derived postbiotics (CFSM) function as effective antivirulence agents that disrupt both the transcriptional networks governing biofilm synthesis and SPI-1-mediated host invasion, while physically compromising bacterial cell wall integrity. These findings highlight probiotic CFSM as a sustainable, non-antibiotic strategy to mitigate PDR *Salmonella* persistence across the food-to-clinical continuum.

## Introduction

Non-typhoidal *Salmonella enterica* (NTS) remains a paramount global public health concern, serving as a primary driver of foodborne gastroenteritis and multi-drug resistant (MDR) infections worldwide (Olubisose et al., 2021; Kumar et al., 2025). Particular serovars, such as *Salmonella enterica* serovar Anatum (*S.* Anatum), along with other high-risk strains, present significant therapeutic challenges due to their increasing resistance profiles (El-Demerdash et al., 2018; Mafe et al., 2025). The persistence of *Salmonella* across the food supply chain is largely driven by its robust biofilm-forming capacity and coordinated virulence machinery, which enable it to withstand antimicrobial stressors in retail meat and successfully invade human intestinal epithelium (Pang et al., 2023; Mi et al., 2024). In many developing regions, however, the epidemiological link between retail meat contamination and human clinical infection remains insufficiently characterized, leaving a critical gap in our understanding of local zoonotic transmission dynamics (Dushayeva, 2025; Mafe et al., 2025).

To overcome escalating antimicrobial resistance, recent investigations have increasingly prioritized non-antibiotic biocontrol strategies, particularly postbiotics derived from lactic acid bacteria and spore-forming probiotics (Amobonye et al., 2025; Pakbin et al., 2026; Parlindungan et al., 2026). While cell-free spent media (CFSM) from probiotic bacteria—such as *Lactobacillus*, *Bifidobacterium*, *Enterococcus*, and *Bacillus* species—have demonstrated general antibacterial activity, the precise transcriptional mechanisms through which these metabolic products silence specific virulence networks remain incompletely understood.

Building directly upon our previous epidemiological characterization of high-risk *Salmonella* serovars in animal-origin food products (El-Demerdash et al., 2021), this study addresses these crucial knowledge gaps through a two-fold approach. First, we adopt a “One Health” cross-sectional design to establish the epidemiological connection between pan-resistant clinical isolates from severe gastroenteritis cases and contaminated retail meat products from the same geographic region. Second, to address the therapeutic need for targeted antivirulence interventions, we investigate the capacity of multi-genus probiotic CFSM preparations to suppress key phenotypic and transcriptomic virulence determinants—specifically target genes governing biofilm formation (*csgD*), quorum sensing (*sdiA*), and *Salmonella* pathogenicity island-1 (SPI-1)-mediated invasion (*hilA*, *invF*, *prgH*, and *sipA*).

## Materials and methods

### Bacterial strains, isolation, and culture conditions

This study utilized *Salmonella enterica* isolates from both food and human clinical sources to ensure a comprehensive assessment of probiotic efficacy.

Animal-Source Isolates: The *Salmonella* strains of animal origin were obtained from our previous epidemiological survey in the Qalyubiyah Governorate, Egypt (El-Demerdash et al., 2021). These included 70 retail meat samples (luncheon, burger, sausage, minced meat, raw meat, canned beef, and kofta), which yielded eight isolates with characterized multidrug resistance profiles. From this collection, two representative pan-antibiotic-resistant serovars -of six isolates- were selected for biocontrol experiments: *Salmonella* Anatum and *Salmonella* Kentucky, originally isolated from minced meat and burger products.

#### Human clinical isolates

Human clinical samples, comprising 10 stool and 10 blood specimens, were prospectively collected from patients presenting with symptoms of acute gastroenteritis at local hospitals and clinical laboratories in the same region and period. *Salmonella* isolation from these clinical samples was performed according to standard microbiological protocols (ISO, 2017; El-Demerdash et al., 2023c). Briefly, samples underwent pre-enrichment and selective enrichment, followed by plating on selective agar media. Presumptive colonies were confirmed as *Salmonella enterica* through biochemical and serological identification.

#### Probiotic strains and growth media

The study evaluated 11 probiotic strains belonging to four distinct genera (*Lactobacillus*, *Bifidobacterium*, *Enterococcus*, and *Bacillus*) to determine their antimicrobial, antibiofilm, and anti-invasion potential. *Enterococcus* and *Bacillus* species were cultured in de Man, Rogosa, and Sharpe (MRS) broth and Nutrient broth, respectively, under microaerophilic conditions (5% O_2_, 10% CO_2_, 85% N_2_) at 37 °C for 24 h. For the cultivation and isolation of *Bifidobacterium* strains, MRS broth was supplemented with 0.05% (w/v) L-cysteine hydrochloride to reduce oxidation-reduction potential, and incubations were maintained under strict anaerobic conditions (85% N_2_, 10% H_2_, 5% CO_2_) using an anaerobic jar system (GasPak EZ, BD Biosciences) at 37 °C for 48 h (Soltani et al., 2022; Hashem et al., 2024; Gómez-Martínez et al., 2026). The full strain identifiers for all probiotics evaluated in this study include *Lactobacillus* species [such as EMCC 1027, ATCC 14917, CCUG 30503], *Bifidobacterium bifidum* [EMCC 1334], and *Enterococcus faecium* [ATCC 19433].

Prior to use in experiments, all *Salmonella* serovars were cultured in Nutrient Broth (Oxoid, Hampshire, UK) at 37 °C for 24 h. Probiotic strains were routinely cultured in de Man, Rogosa, and Sharpe (MRS; Oxoid) broth at 37 °C for 24 h under appropriate atmospheric conditions.

### Phenotypic antimicrobial susceptibility testing and PDR characterization

To rigorously characterize the multi-drug and pan-drug resistance (PDR) profiles of the *Salmonella* isolates, minimum inhibitory concentrations (MICs) were determined using the broth microdilution method according to Clinical and Laboratory Standards Institute (CLSI M100-ED33) guidelines. Nine representative antibiotic classes were tested: aminoglycosides (gentamicin), β-lactams (ampicillin), β-lactam/lactamase inhibitor combinations (amoxicillin-clavulanic acid), extended-spectrum cephalosporins (ceftriaxone, cefotaxime), carbapenems (meropenem), fluoroquinolones (ciprofloxacin), folate pathway antagonists (trimethoprim-sulfamethoxazole), phenicols (chloramphenicol), and tetracyclines (tetracycline). Testing was performed in Mueller-Hinton broth (MHB; Oxoid) with an initial inoculum of 5×10^5^ CFU/mL. Plates were incubated at 35 ± 2^∘^ C for 18–24 h. Strains were categorized as PDR based on non-susceptibility to all tested agents across all nine antimicrobial categories.

### Molecular confirmation of *Salmonella enterica* isolates

Genotypic identification of *Salmonella enterica* isolates was performed using genus-specific PCR targeting a 284 bp fragment of the *invA* gene, employing forward (5′-GTG AAA TTA TCG CCA CGT TCG GGC AA-3′) and reverse (5′-TCA TCG CAC CGT CAA AGG AAC C-3′) primers (Upadhyay et al., 2010). Genomic DNA was extracted using a QIAamp DNA Mini Kit (Qiagen, Germany) according to the manufacturer’s instructions. A reference strain of *Salmonella enterica* subsp. *enterica* serovar Typhimurium (ATCC 14028) served as the positive control, while nuclease-free water replaced template DNA in the non-template negative control (NTC).

Amplification was carried out in a conventional thermal cycler under the following conditions: initial denaturation at 94 °C for 1 min; followed by 35 cycles of denaturation at 94 °C for 1 min, annealing at 58.3 °C for 30 s, and extension at 72 °C for 30 s; with a final extension at 72 °C for 7 min. PCR products were mixed with gel loading buffer and resolved by electrophoresis on a 1.5% (w/v) agarose gel stained with ethidium bromide (0.5 µg/mL) in 1× TAE buffer at a constant voltage of 100 V for 40 min. A 100 bp DNA ladder (Qiagen, Germany) was included as a molecular weight marker. Amplified DNA fragments were visualized and photographed under UV transillumination.

### Preparation of probiotic cell-free spent media

To evaluate the antimicrobial, antibiofilm, and antivirulence potential of the probiotic candidates, cell-free spent media (CFSM) were prepared following established protocols (Abdelhamid et al., 2018; Mohamed et al., 2020) with minor modifications.

#### Broth-based CFSM preparation

The 11 probiotic strains (representing *Lactobacillus, Bifidobacterium, Enterococcus*, and *Bacillus* genera) were initially cultured in MRS broth for 18 h at 37 °C under 5% CO_2_. For CFSM extraction, overnight cultures were inoculated (1:100 dilution) into fresh MRS broth and incubated under the same conditions until reaching a standardized optical density (OD_600_) of 1.6. The cultures were then centrifuged at 4,000 × g for 10 min at 4 °C. The resulting supernatants were collected and filter-sterilized using 0.22 µm pore-size membranes (Millipore, USA) to ensure the complete removal of bacterial cells.

#### Milk-based CFSM preparation (fermented milk)

To assess activity in a food-matrix simulant, reconstituted skim milk (Nestlé, Cairo, Egypt) was prepared by heating at 65 °C for 30 min, followed by overnight cooling at 4 °C. Prior to inoculation, the sterile milk matrix was equilibrated to 37^∘^C in a temperature-controlled water bath. The milk was inoculated with the probiotic strains and incubated under microaerophilic conditions at 37 °C for 24 h. The fermented milk CFSM was harvested via centrifugation at 4,000 × g for 15 min at 15 °C. The supernatant was subsequently filter-sterilized as described above (Abdelhamid et al., 2018).

All CFSM preparations were aliquoted and stored at −20 °C to maintain the stability of bioactive metabolites for downstream antimicrobial and gene expression assays.

### Mechanistic characterization and pH neutralization controls of CFSM

To distinguish whether the antimicrobial, antibiofilm, and gene-modifying effects were driven by organic acidity (low pH) or by bioactive postbiotic metabolites (such as bacteriocins, bio-surfactants, organic acids in non-protonated forms), two comparative control groups were incorporated into all downstream assays:

1. pH-Neutralized CFSM (CFSM_neut_): Aliquots of crude CFSM were adjusted to pH 6.5 ± 0.1 using 1M NaOH to eliminate free-acid-mediated inhibitory activity.
2. Acidic Medium Control (MRS_acid_): Uninoculated MRS broth was adjusted with L-lactic acid (Sigma-Aldrich) to match the exact pH of the crude CFSM (pH 3.8 - 4.2).

### Determination of antibacterial activity

#### Agar well diffusion assay

The antibacterial potential of crude, pH-neutralized, and acidic-control CFSM preparations against the Salmonella serovars was evaluated using a modified agar well diffusion assay (Fredua-Agyeman and Gaisford, 2019; Al-Shemy et al., 2025). To prepare the indicator strains, *S. enterica* serovars were cultured on Trypticase Soy Agar (TSA; Oxoid) for 24 h at 37 °C. Individual colonies were then inoculated into Trypticase Soy Broth (TSB; Oxoid) and incubated for 24 h at 37 °C to reach a standardized inoculum density.

For the diffusion assay, sterile cotton swabs were saturated with the *Salmonella* cultures and used to inoculate the surface of TSA plates in three directions to ensure a confluent lawn. Five equidistant wells (6 mm diameter) were punched into the agar using a sterile cork borer and filled with 100 µl of the respective probiotic CFSM. After aerobic incubation at 37 °C for 24 h, the diameters of the zones of inhibition (ZOI) were measured. A ZOI ≥10 mm was categorized as positive inhibitory activity.

#### Determination of MIC and SIC via broth microdilution

To precisely quantify the potency of the CFSM and identify non-lethal concentrations for genetic analysis, the Minimum Inhibitory Concentration (MIC) and Sub-Inhibitory Concentration (SIC) were determined using the broth microdilution method in 96-well microtiter plates (CLSI, 2023; Amin et al., 2025).

Briefly, the probiotic CFSM was two-fold serially diluted in TSB to obtain a concentration range from 50% to 0.39% (v/v). Each well was then inoculated with a standardized *Salmonella* suspension to achieve a final concentration of approximately 1 x 10^6^ CFU/mL. The plates were incubated at 37 °C for 24 h. The MIC was defined as the lowest concentration of CFSM that completely inhibited visible bacterial growth.

The SIC was identified as the highest concentration of CFSM that did not significantly affect the growth kinetics or viability of the *Salmonella* serovars compared to the untreated control. This SIC (determined to be 10% v/v based on growth curve analysis) was subsequently utilized for all biofilm reduction and gene expression (RT-qPCR) assays to ensure that observed effects were due to the attenuation of virulence rather than bactericidal action.

### Biofilm inhibition and quantification assay

Biofilm inhibition was evaluated using the crystal violet assay (Medellin-Peña and Griffiths, 2009; Sewid et al., 2025). Exponential-phase Salmonella cultures (180 µL) were seeded into 96-well microtiter plates containing 20 µL (10% v/v final concentration) of crude CFSM, pH-neutralized CFSM, MRS _acid_ control, or sterile broth (untreated control). Plates were incubated at 37 °C for 16 h. Planktonic cells were aspirated, wells washed thrice with distilled water, air-dried, and stained with 150 µl of 0.1% (w/v) crystal violet for 30 min. Bound dye was solubilized and quantified at OD_595_ using a microplate reader (Sunrise, TECAN Inc., USA). Biofilm reduction potency was calculated relative to the untreated control using the following formula:


$$\text{Biofilm Inhibition}(\%)=(({\text{OD}}_{\text{Untreated Control}}-{\text{OD}}_{\text{Treated}})/{\text{OD}}_{\text{Untreated Control}})\times 100$$

### *In vitro* invasion assay (gentamicin protection assay)

To evaluate the impact of probiotic cell-free spent medium (CFSM) on the invasive capacity of *Salmonella* serovars, a gentamicin protection assay (Elsinghorst, 1994) was performed using human intestinal epithelial (Caco-2) cells. These cells are maintained in Dulbecco’s Modified Eagle Medium (DMEM) supplemented with 10% fetal bovine serum (FBS) and 1% non-essential amino acids, then seeded into 24-well plates and incubated at 37 °C under 5% CO_2_ until a confluent monolayer of approximately 1 x10^5^ cells/well is achieved for exactly 1 hour prior to infection.

*Salmonella* cultures were grown to the exponential phase and standardized to a concentration of 1 x 10^8^ CFU/mL. The Caco-2 monolayers are rinsed with phosphate-buffered saline (PBS) and subsequently infected at a multiplicity of infection (MOI) of 10:1 using either untreated *Salmonella* as a control or *Salmonella* pre-exposed to a 10% (v/v) sub-inhibitory concentration (SIC) of crude CFSM, pH-neutralized CFSM, or control media. Following a 2 h incubation period at 37 °C to permit bacterial internalization, the extracellular, non-adherent bacteria are removed by washing the monolayers three times with PBS. Any remaining extracellular bacteria are then eliminated by treating the wells with DMEM containing a high concentration of gentamicin (100 µg/mL) for 1 h.

Following gentamicin treatment, the monolayers are washed again and lysed with 0.1% Triton X-100 to release the internalized bacteria. The resulting lysates are serially diluted and plated onto TSA for colony enumeration. The invasion efficiency is determined by calculating the percentage of the initial bacterial inoculum that remained viable following gentamicin treatment.

Inhibition of invasion % = 100(1 - TB/TL), where TB and TL are the numbers of invading bacterial cells (log CFU/well) in the presence and absence of treatment, respectively (Ammar et al., 2021).

### RNA extraction and RT-qPCR analysis of virulence gene expression

To investigate the molecular mechanisms underlying the antivirulence activity of probiotic postbiotics, the expression levels of key biofilm and invasion genes were quantified using Reverse-Transcription Quantitative PCR (RT-qPCR).

To evaluate non-lethal transcriptomic silencing without impairing cell viability, *Salmonella* isolates were exposed to a experimentally verified non-lethal 10% (v/v) sub-inhibitory concentration (SIC) of crude (acidic, pH 4.0 ± 0.1) MRS-derived CFSM for 16 h at 37 °C. Untreated *Salmonella* cultures in standard broth served as the baseline control.

The *Salmonella* isolates were cultured in nutrient broth supplemented with a 10% (v/v) sublethal concentration of MRS-derived CFSM; untreated cultures served as the control. Following incubation at 37 °C for 24 h, the cells were harvested via centrifugation. To maintain the integrity of the transcriptional profiles, an appropriate volume of RNAprotect Bacteria Reagent (Qiagen, Hilden, Germany) was immediately added to the cell pellets. The mixture was vortexed and stabilized for 5 min at room temperature before being centrifuged at 8,000 rpm for 10 min.

Total RNA was extracted from the stabilized cell pellets using the RNeasy Mini Kit (Qiagen, Hilden, Germany) according to the manufacturer’s instructions. RNA concentration, purity (A260/A280 ratio ≥ 1.9), and structural integrity were verified spectrophotometrically (NanoDrop 2000, Thermo Fisher Scientific, USA) prior to downstream applications.

The RT-qPCR reactions were performed in a total volume of 25 µl using the StepOne™ Real-Time PCR System. Each reaction mixture contained 12.5 µl of 2x QuantiTect SYBR Green PCR Master Mix (Qiagen), 0.25 µl of RevertAid Reverse Transcriptase (200 U/µL; Thermo Fisher Scientific), 0.5 µl of each forward and reverse primer (20 pmol), and 5 µl of the RNA template (balanced with nuclease-free water). Novel primer sets were specifically engineered using Primer3 and FastPCR software to ensure high specificity and optimal melting temperatures. These designs were further validated through experimental optimization to confirm their performance for the targeted gene sequences (**Table 1**).

**Table 1:** Primers sequences of target genes and cycling conditions for reverse-transcription quantitative PCR used in the current study.

Target gene	Primers sequences (5′ → 3′)	Amplified segment (bp)	Annealing (°C)	Accession no.
16S rRNA	F: TGGCGCATACAAAGAGAAGC	192	55	LC773422.1
	R: TTTTGCAACCCACTCCCATG			
*sdiA*	F: TAGGTAAACGAGGAGCAGCG	187	60	MK431385.1 AF458099.1 MG242331.1
	R: CGGAAAATTTCAGGCAGGGT			
*sipA*	F: AAGTGTAAGGACTCAGCCCC	165	60	
	R: ATCAGCGCGGGAAAATCTTC			
*csgD*	F: CCGCATTACTCACTCATCGC	175	60	
	R: TTTGCCCATGAAACTGCCTG			
*invF*	F: GTAAACGCTGCGAGTACTGG	151	60	U08280.1 AY956822.1
	R: GCGTTCGCTCCAACATGTT			
*prgH*	F: TGGCGGCGTATCAGAGTAAG	168	60	
	R: TAACGGCATTCTCAGGGACA			
*hilA*	F: ATGGCGCAAATGGGGATTTT	158	60	MT130451.1
	R: AGCAAACTCCCGACGATGTA			

The cycling conditions were managed by the StepOne™ software, which provided the amplification curves and cycle threshold (C_T_) values. The 16S rRNA gene was utilized as the internal housekeeping gene for normalization. Relative changes in the expression of target virulence genes were calculated using the 2^-ΔΔCT^ method (Livak and Schmittgen, 2001; El-Demerdash et al., 2023b) according to the following equation:


Fold Change=2−[CT of target gene−CT of housekeeping gene]treated group−[CT of target gene−CT of housekeeping gene]control group

Fold-change values < 1.0 were interpreted as significant gene downregulation relative to the untreated baseline control (1.0), demonstrating the comprehensive molecular silencing capacity of the crude postbiotic fermentate.

### Transmission electron microscopy

Ultra-structural alterations in *Salmonella enterica* serovars treated with crude CFSM were evaluated using Transmission Electron Microscopy (TEM). Bacterial cells (10^8^ CFU/mL) exposed to 10% SIC CFSM for 12 h at 37 °C were harvested by centrifugation (4,000 × g, 10 min) and fixed in 2.5% (v/v) glutaraldehyde in 0.1 M phosphate-buffered saline (PBS, pH 7.2) for 4 h at 4 °C. Samples were post-fixed in 1% (w/v) osmium tetroxide, dehydrated through a graded ethanol series (30%–100%), and embedded in epoxy resin. Ultra-thin sections (70 nm) were cut using an ultramicrotome, stained with uranyl acetate and lead citrate, and examined using a Transmission Electron Microscope (JEOL JEM-1400) operated at 80 kV.

### High-performance liquid chromatography profiling of organic acids

The organic acid profile of the cell-free spent medium (CFSM) derived from the most effective probiotic was characterized and quantified using High-Performance Liquid Chromatography (HPLC) according to previously described protocols with minor modifications (Zhang et al., 2023). Chromatographic separation was performed on an Agilent 1260 Series HPLC system (Agilent Technologies, USA) equipped with a reversed-phase C18 column (4.6 mm x 250 mm i.d., 5 μm particle size).

The mobile phase consisted of 0.2 mM sodium dihydrogen phosphate NaH_2_PO_4_ buffer containing 5% (v/v) acetonitrile, delivered at an isocratic flow rate of 1.0 ml/min. The column temperature was maintained at 30 °C. Samples were filtered through 0.22 μm membrane filters prior to injection, and an injection volume of 10 μL was introduced using a 50 μL syringe assembly. Organic acid detection was monitored at a UV wavelength of 210 nm. Individual organic acids were identified and quantified by comparing their retention times (t_R_) and peak areas (mAU) against authentic organic acid standards.

### Statistical analysis

All data were analyzed using the SPSS Inc. version 26 (IBM Corp., Armonk, NY, USA). One-way ANOVA and Tukey’s *post hoc* tests were used to test the effect of *Lactobacillus helveticus* CFSM and *L. plantarum CFSM* on *Salmonella* virulence genes’ expression and invasion. The normality and homogeneity of variance among the data were determined utilizing Shapiro–Wilk’s and Levene’s tests, respectively. All experimental procedures were done in triplicate. The results were expressed as mean± standard error of the mean (SEM), and the significance level was set at *p* < 0.05. All graphs were made by GraphPad Prism software version 8 (San Diego, CA, USA) and R software version 4.3.3 (https://www.r-project.org/).

## Results

### Selection and AST characterization of pan-drug resistant isolates

To establish a rigorous baseline using extensively antibiotic-resistant pathogens, three clinical *Salmonella* Enteritidis isolates were prospectively recovered from human stool/blood samples, and six meat-associated isolates (3 *S.* Anatum and 3 *S.* Kentucky) were selected from a prior surveillance framework (El-Demerdash et al., 2021). Phenotypic Antimicrobial Susceptibility Testing (AST) via broth microdilution—conducted in strict accordance with CLSI (M100-ED33) guidelines—confirmed that all nine panel isolates exhibited true Pan-Drug Resistance (PDR). As detailed in **Supplementary Table S1**, every isolate demonstrated complete resistance (MICs well exceeding clinical susceptibility breakpoints) across all nine tested antimicrobial categories, including extended-spectrum cephalosporins (ceftriaxone MIC ≥ 64 µg/mL), carbapenems (meropenem MIC ≥ 16 µg/mL), fluoroquinolones (ciprofloxacin MIC ≥ 8µg/mL), and aminoglycosides (gentamicin MIC ≥ 32 µg/mL).

### Molecular identification of pan-drug resistant *Salmonella* isolates

Polymerase chain reaction (PCR) amplification of the genus-specific invasion gene (*invA*) was conducted to molecularly confirm the identity of nine phenotypically identified pan-drug resistant (PDR) bacterial isolates. All nine PDR isolates (100%) yielded a single, distinct, and well-defined amplicons corresponding to the predicted target size of the *invA* gene (284 bp, **Supplementary Figure S1**).

### Antimicrobial potency and mechanistic dissection of probiotic CFSM

Evaluating the inhibitory potential of CFSMs revealed a marked dependency on growth substrate. Probiotics cultured in MRS broth yielded significantly larger inhibition zones than those grown in reconstituted skim milk (**Figure 1**, **2**). Among MRS-derived preparations, crude Lactobacillus helveticus EMCC 1654 CFSM exhibited the most potent bactericidal activity (25–40 mm zones of inhibition), followed closely by *Lactobacillus plantarum* CCUG 30503. *S.* Anatum was the most sensitive pathogen, whereas *S.* Kentucky proved the most resilient.

**Figure 1:**
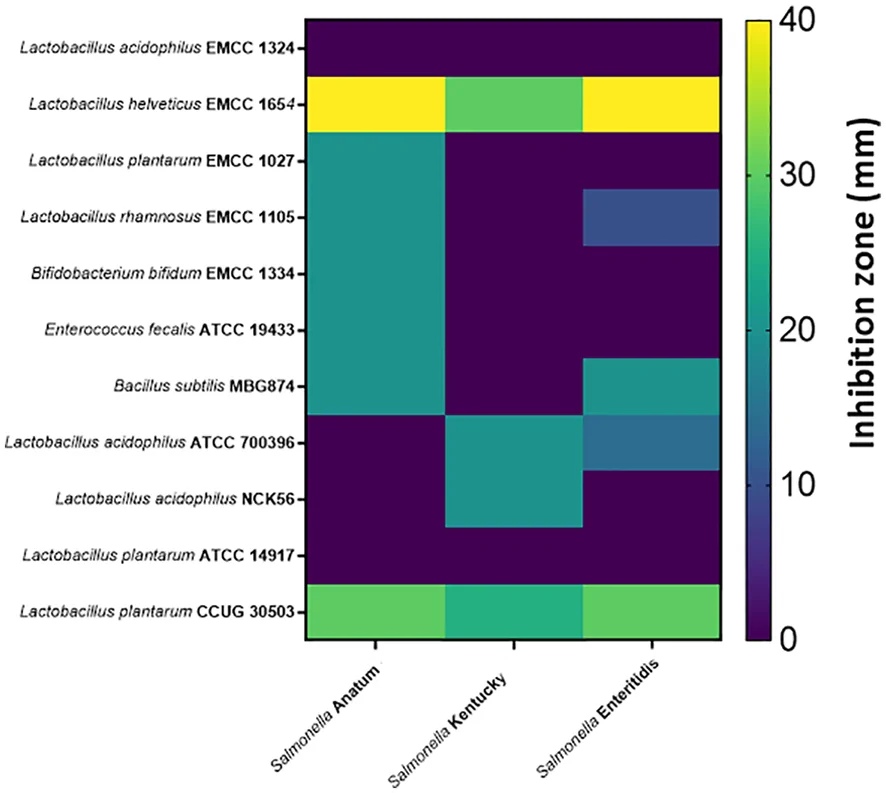
Heatmap illustrating antimicrobial activity (expressed as diameters in millimeters of inhibition zones) of cell-free preparations of probiotic strains grown in MRS broth against pan *Salmonella enterica* serovars.

**Figure 2:**
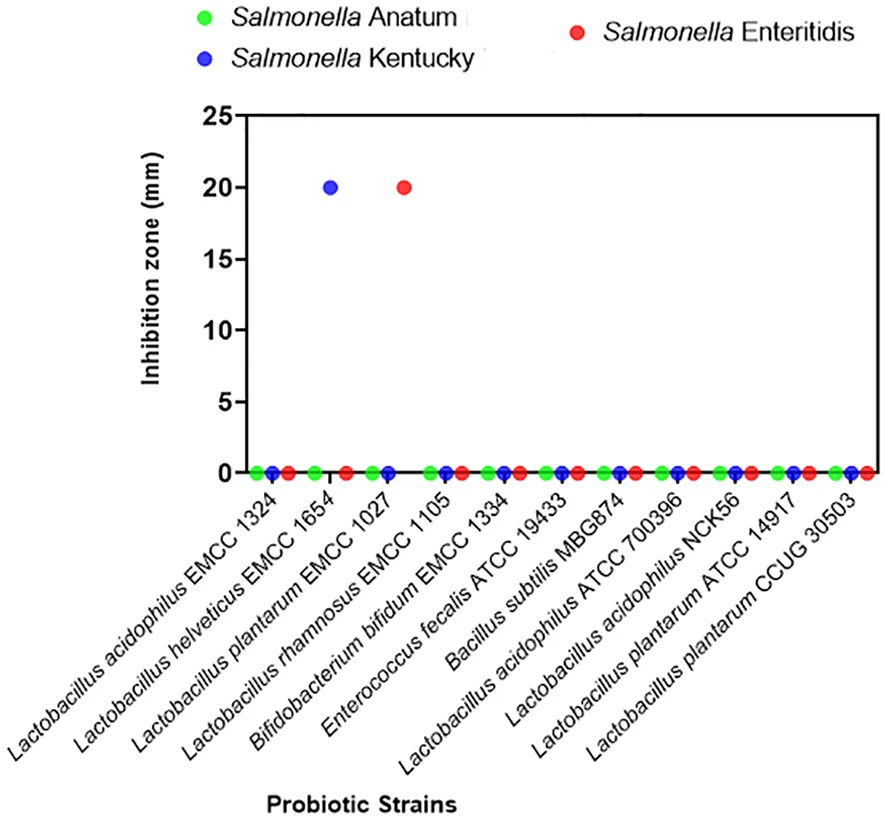
Antimicrobial activity (expressed as diameters in millimeters of inhibition zones) of cell-free preparations of probiotic strains grown in reconstituted skim milk against pan *Salmonella enterica* serovars isolated.

To address whether direct growth inhibition was driven strictly by organic acidity or by bioactive postbiotic compounds:

- Crude CFSM (pH 3.8 - 4.2): Produced maximum zones of inhibition against all three serovars (25–40 mm). pH-Neutralized CFSM (CFSM_neut_, pH 6.5): Inhibitory zones on agar diffusion were completely abolished or drastically reduced (0–8 mm), indicating that bactericidal/growth-inhibitory pressure relies heavily on protonated organic acids.
- Acidic Medium Control (MRS_acid_, pH 4.0): Produced moderate zones of inhibition (12–18 mm), confirming that acidity alone accounts for partial antibacterial activity, but lacks the full potency of the crude fermentate.

### Probiotic CFSM attenuates biofilm formation independent of pH

In contrast to agar growth inhibition, antibiofilm assays performed at sub-inhibitory concentrations (10% v/v SIC) demonstrated that non-acidic postbiotics actively disrupt biofilm architecture.

- Crude CFSM: Exhibited the strongest biofilm reduction (78%- 92% reduction across serovars; p < 0.0001).
- pH-Neutralized CFSM (CFSM_neut_): Retained a significant, high level of biofilm attenuation (52%-71% reduction; p < 0.001).
- MRS_acid_ Control: Produced only a minor reduction in biofilm mass (15%-24%).

These results confirm that while organic acids contribute to overall biomass reduction, bioactive molecules (such as bacteriocins, biosurfactants) present in neutralized CFSM actively interfere with the extracellular polymeric substance (EPS) matrix and cell attachment mechanisms.

### Attenuation of *Salmonella* invasion efficiency in caco-2 cells via probiotic CFSM

The Gentamicin protection assay demonstrated that pre-treatment with probiotic cell-free spent media (Crude CFSM) at a 10% (v/v) sub-inhibitory concentration (SIC) significantly impaired the invasive capacity of pan-drug resistant (PDR) *Salmonella* serovars (p < 0.0001). For all tested isolates, Crude CFSM treatment resulted in a multi-log reduction of internalized bacteria within human Caco-2 intestinal epithelial cells compared to untreated controls (**Figure 3**; **Supplementary Table S2**).

**Figure 3:**
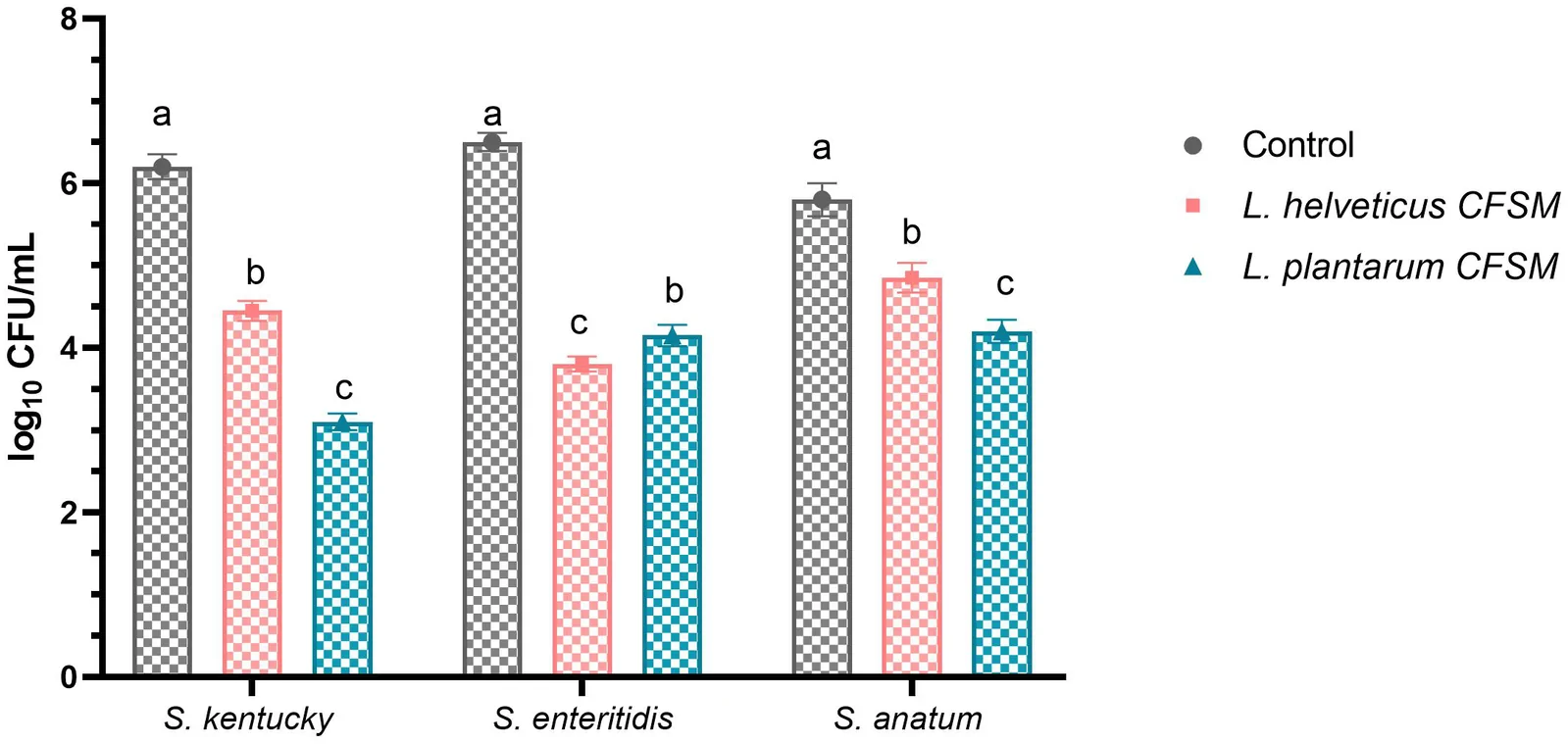
Effects of *L. helveticus* CFSM and *L. plantarum* treatments on *Salmonella* invasion of Caco-2 cells. Results were the mean of three independent experiments ± standard error of the mean (SEM) in bars. ^a-c^ Means with different superscript letters differ significantly at *p* < 0.05.

The most profound attenuation was observed in *S.* Kentucky treated with *L. plantarum* CFSM. The intracellular bacterial load was reduced from 6.20 ± 0.15 Log_10_ CFU/mL (control) to 3.10 ± 0.10 Log_10_ CFU/mL (p < 0.05). This 3.1-log reduction corresponds to a 50% inhibition of the baseline invasion (**Figure 4**). When treated with *L. helveticus*, *S.* Kentucky also showed a significant decrease in internalized counts to 4.45 ± 0.12 Log_10_ CFU/mL.

**Figure 4:**
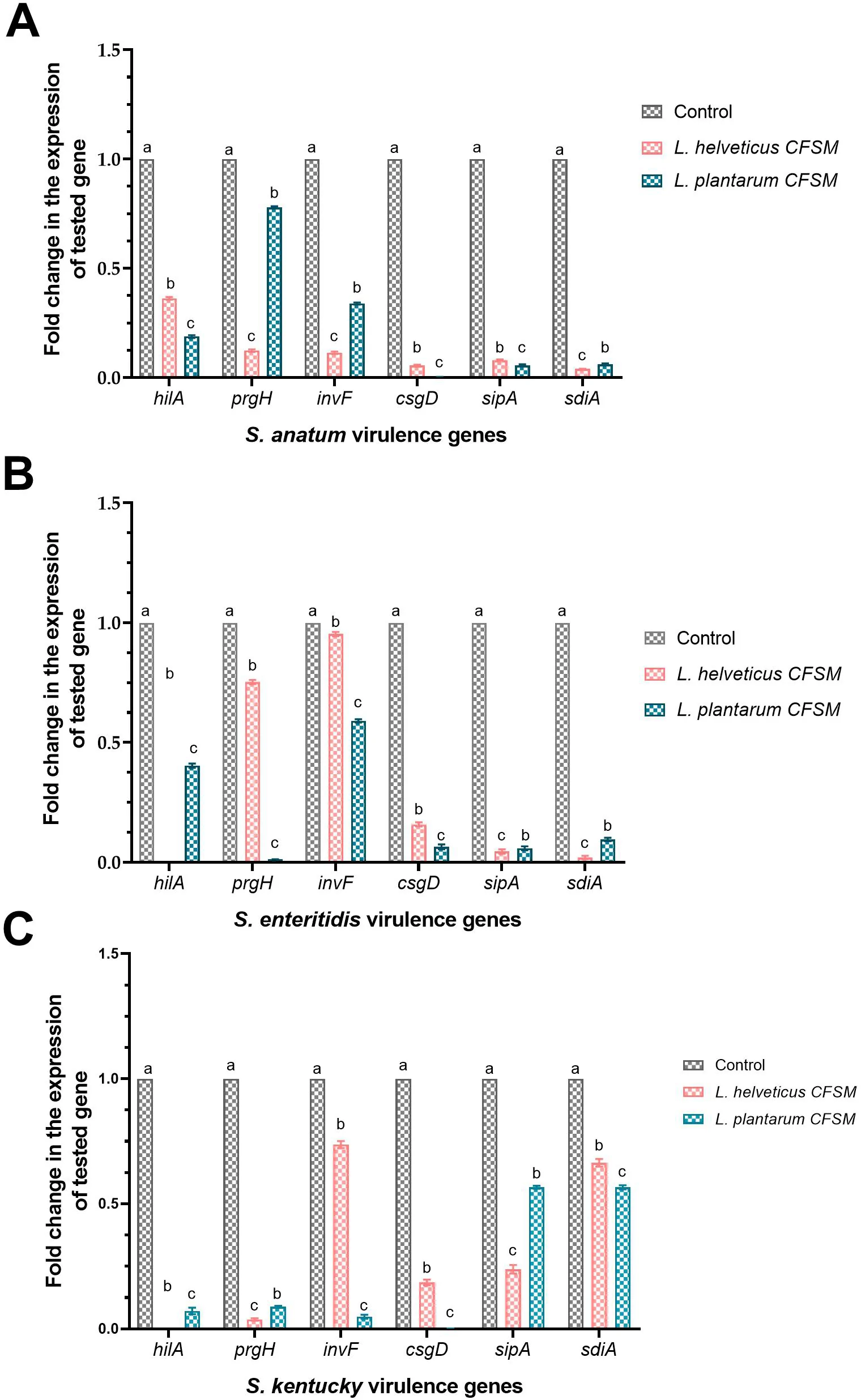
Fold changes in the expressions of *hilA, prgH, invF, csgD, sipA*, and *sdiA* virulence genes among *Salmonella anatum* **(A)**, *S. enteritidis* **(B)**, and *S. kentucky* **(C)** isolates after treatment with *Lactobacillus helveticus* CFSM and *Lactobacillus plantarum* CFSM. Values represent the fold changes in comparison with the transcription levels of the control untreated isolates, which were assigned a value of 1. Results were expressed as mean of three independent experiments± standard error of the mean (SEM) in bars. ^a-c^ Means with different superscript letters differ significantly at *p* < 0.05.

The clinical isolate *S.* Enteritidis exhibited the highest baseline invasion (6.50 ± 0.11 Log_10_ CFU/mL). Treatment with *L. helveticus* CFSM was particularly effective, reducing internalized counts to 3.80 ± 0.09 Log_10_ CFU/mL, achieving a relative invasion of 58.46± 1.38 compared to the control (p < 0.05). Similarly, *L. plantarum* CFSM reduced *S.* Enteritidis invasion to 4.15 ± 0.13 Log_10_ CFU/mL.

For the meat-source isolate *S.* Anatum, pre-treatment with *L. plantarum* CFSM resulted in a reduction of the intracellular load to 4.20 ± 0.14 Log_10_ CFU/mL (p < 0.05). Notably, *S.* Anatum treated with *L. helveticus* showed a higher recovery of internalized cells (4.85 ± 0.18 Log_10_ CFU/mL) compared to other treatments, though this still represented a significant reduction from the untreated control (5.80 ± 0.20 Log_10_ CFU/mL).

These findings, characterized by distinct superscript letters (^a-c^) within each serovar group, indicate that while all CFSM treatments significantly diminished pathogenicity, the degree of attenuation is highly serovar-dependent and probiotic strain-specific.

Crucially, testing pre-exposure with CFSM_neut_ yielded a 1.8-2.4-log reduction in internalized bacteria without altering pathogen viability during the pre-exposure phase, whereas MRS_acid_ controls resulted in less than a 0.5-log reduction.

### Molecular attenuation of *Salmonella* virulence through gene silencing

The RT-qPCR analysis revealed that pre-treatment with *L. helveticus* and *L. plantarum* CFSM induced a statistically significant downregulation across all six target virulence genes (p < 0.0001) in *S.* Anatum, *S.* Enteritidis, and *S.* Kentucky (**Figure 4**). Using a standardized baseline of 1.0 for untreated controls, the probiotic postbiotics effectively transitioned the pathogens into an attenuated state.

#### Suppression of the SPI-1 invasion machinery

The CFSM treatments targeted the core components of the Salmonella Pathogenicity Island 1 (SPI-1), which is essential for host cell entry.

- The Master Switch (*hilA*): *L. plantarum* exerted the strongest suppression on the central regulator *hilA* in *S.* Kentucky (0.071) and *S.* Anatum (0.188).
- The Structural Needle (*prgH* & *invF*): In *S.* Enteritidis, *L. plantarum* nearly abolished the expression of *prgH* (0.012), a key component of the Type III Secretion System (T3SS) baseplate. Similarly, *invF*—the gatekeeper for effector secretion—was most severely silenced in *S.* Kentucky (0.048) and *S.* Anatum (0.113) (**Supplementary Tables S3**-**S5**).
- The Actin Remodeler (*sipA*): The gene encoding the actin-binding protein *sipA*, which mediates membrane ruffling for intracellular entry, was significantly downregulated across all serovars. The most notable reduction occurred in *S.* Enteritidis treated with *L. helveticus* (0.045).

#### Inhibition of biofilm and quorum sensing regulators

The probiotic preparations also targeted the genetic drivers of environmental persistence and cell-to-cell communication.

- The Biofilm Master Regulator (*csgD*): *L. plantarum* exhibited an extraordinary ability to silence *csgD*, the activator of curli fimbriae. In *S.* Kentucky, expression dropped to an almost undetectable 0.001, and in *S.* Anatum to 0.002, effectively neutralizing the bacteria’s ability to construct a protective biofilm matrix.
- Quorum Sensing Quenching (*sdiA*): The *sdiA* gene, which allows *Salmonella* to sense signals from other bacterial species, was consistently suppressed. *L. helveticus* was particularly effective against the clinical *S.* Enteritidis isolate, reducing *sdiA* expression to 0.020.

#### Comparative efficacy and heatmap analysis

As illustrated in the Heatmap (**Figure 5**), while both probiotic strains are potent “antivirulence” agents, their efficacy is serovar-dependent. *L. plantarum* showed a specialized potency in silencing biofilm-related genes (*csgD*) in the meat-source isolates (*S.* Kentucky and *S.* Anatum), whereas *L. helveticus* showed superior performance in suppressing quorum sensing (*sdiA*) and invasion effectors (*sipA*) in the clinical *S.* Enteritidis strains.

**Figure 5:**
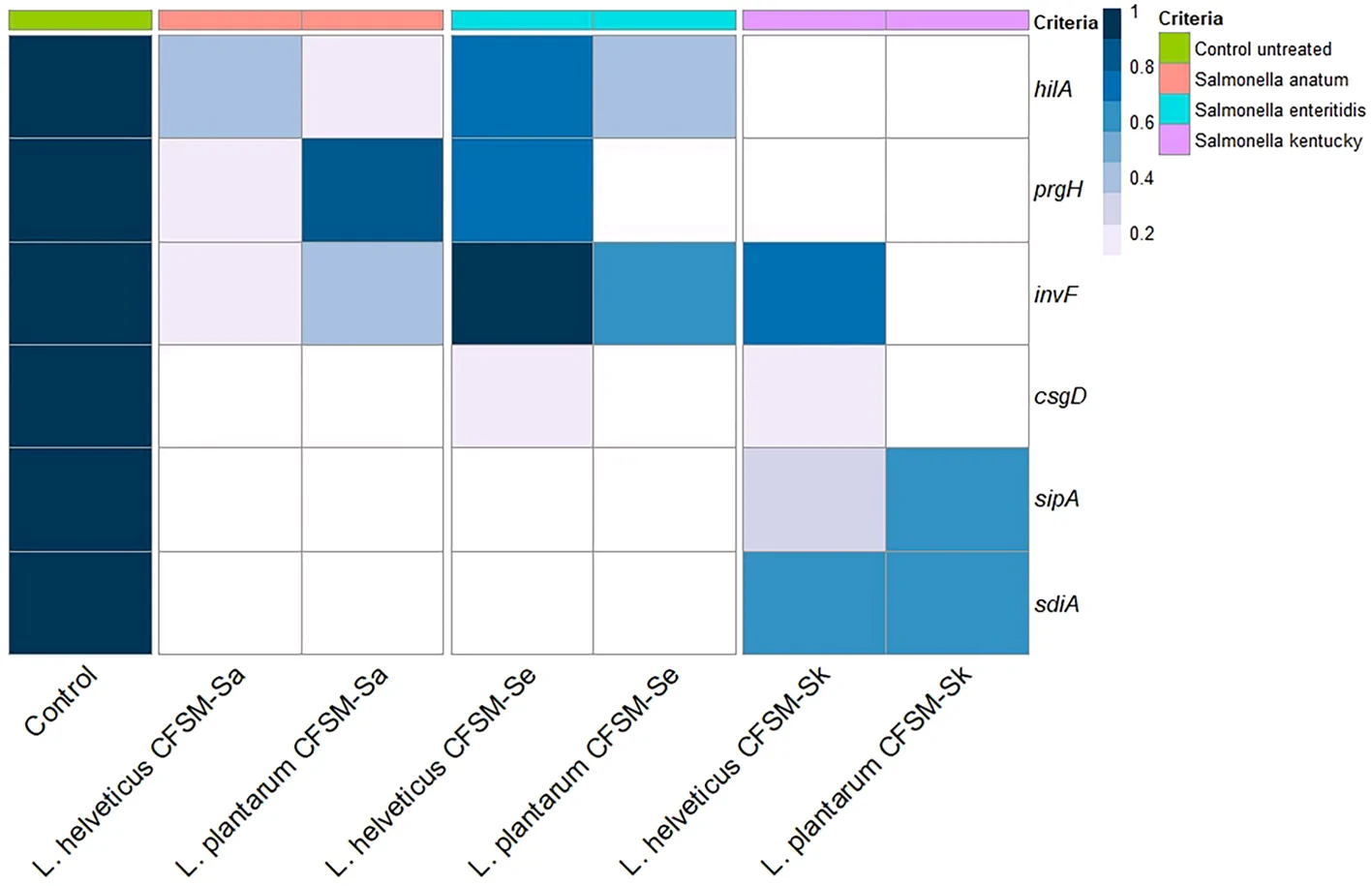
Modulation of *Salmonella* virulence gene expression by *Lactobacillus* treatments. Heatmap displaying fold changes in *hilA*, *prgH*, *invF*, *csgD*, *sipA*, and *sdiA* expression among *Salmonella* Anatum, *S.* Enteritidis, and *S.* Kentucky isolates following treatment with *Lactobacillus helveticus* CFSM or *Lactobacillus plantarum* CFSM. The scale bar on the right indicates fold-change values.

Collectively, these functional data validate the “molecular disarming” hypothesis. The physical failure of PDR *Salmonella* to penetrate the intestinal monolayer (as shown in the invasion assays) provides a direct link between the transcriptional silencing of SPI-1 genes and the potential for these probiotic postbiotics to mitigate the severity of zoonotic infections.

### Ultrastructural changes in *Salmonella* induced by crude CFSM extracts

Transmission electron microscopy (TEM) was performed to evaluate the ultrastructural alterations and membrane-disruptive effects on *Salmonella enterica* subsp. *enterica* serovar Kentucky following treatment with sub-inhibitory concentrations (10% SIC) of crude cell-free spent medium (CFSM) extracts from *Lactobacillus plantarum* and *Lactobacillus helveticus* (**Figure 6**).

**Figure 6:**
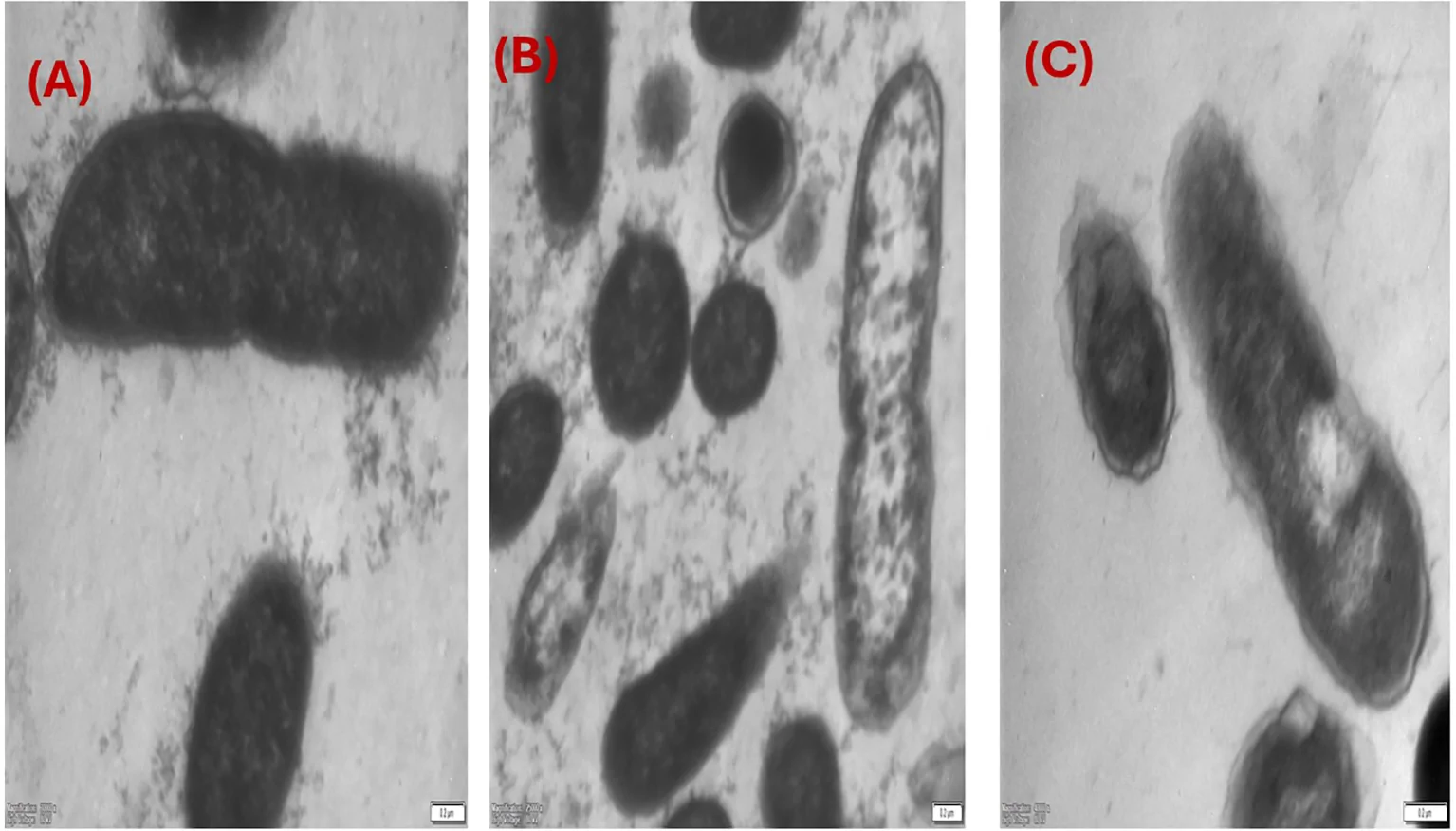
Transmission electron micrographs (TEM) showing the ultrastructural changes in *Salmonella enterica* subsp. *enterica* serovar Anatum following treatment with crude cell-free supernatant matrix (CFSM) extracts. **(A)** Untreated control *Salmonella* Anatum displaying an intact cell wall and dense cytoplasm (30,000x). **(B)** *Salmonella* Anatum treated with *Lactobacillus plantarum* CFSM showing membrane distortion and partial cytoplasmic loss (25,000x). **(C)** *Salmonella* Anatum treated with *Lactobacillus helveticus* CFSM demonstrating severe envelope disruption, internal vacuolation/lesions, and cellular lysis (40,000x). Scale bar = 0.2 µm.

- Control (**Figure 6A**): Untreated *Salmonella* Kentucky cells exhibited typical, intact rod-shaped morphology characterized by continuous, well-defined double-membrane envelopes, smooth outer surfaces, and dense, homogeneously distributed cytoplasm without visible structural defects.
- *Lactobacillus plantarum* CFSM Treatment (**Figure 6B**): Exposure to *L. plantarum* CFSM induced noticeable cell envelope modifications, including outer membrane rippling, distorted cell outlines, localized periplasmic space expansion, and cytoplasmic clearing, indicating early membrane compromise.
- *Lactobacillus helveticus* CFSM Treatment (**Figure 6C**): Exposure to *L. helveticus* CFSM resulted in severe ultrastructural damage. Micrographs displayed pronounced membrane detachment and wrinkling, prominent internal electron-lucent lesions (vacuolation), and extensive loss of intracellular contents leading to cellular lysis.

These morphological findings demonstrate that crude CFSM extracts from both *Lactobacillus* species compromise the structural integrity of the *Salmonella* Kentucky envelope, with *L. helveticus* exerting a markedly higher degree of cell membrane lysis.

### Quantitative organic acid profiling via HPLC

HPLC profiling of the postbiotic fraction derived from *L. plantarum* CCUG 30503 revealed a multi-component organic acid signature dominated by primary fermentation end-products alongside metabolic intermediates (**Figure 7**; **Table 2**).

**Figure 7:**
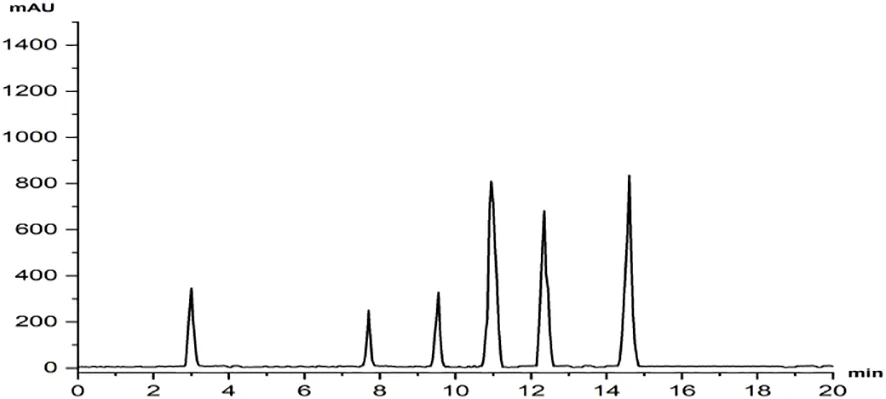
High-Performance Liquid Chromatography (HPLC) chromatogram of organic acids identified in the cell-free spent medium (CFSM) of *Lactobacillus plantarum* CCUG 30503 at a UV wavelength of 210 nm.

**Table 2:** HPLC quantification of organic acid components in *Lactobacillus plantarum* CCUG 30503 cell-free spent medium (CFSM).

Peak No.	Retention time (min)	Identified compound	Peak area (mAU)	Concentration (mg/mL)
1	3.00	Oxalic acid	52.33	0.47
2	7.70	Malic acid	27.08	0.30
3	9.55	Glycolic acid	42.53	0.43
4	10.95	Lactic acid	184.78	1.89
5	12.35	Acetic acid	131.13	1.11
6	14.60	Succinic acid	155.63	1.53

Lactic acid emerged as the predominant metabolite in the cell-free spent medium, accounting for the highest chromatographic peak intensity and constituting the primary driving factor behind CFSM-mediated acidification. This was accompanied by substantial levels of succinic and acetic acids, which together formed the core tricarboxylic acid and short-chain organic acid pool generated during probiotic growth.

In addition to these major metabolites, smaller quantitative fractions of dicarboxylic and hydroxy acids—specifically oxalic, glycolic, and malic acids—were resolved at earlier retention times. The simultaneous presence of these minor acids complements the primary lactic and acetic acid pool, reflecting an active metabolic flux that collectively contributes to the physiological and antimicrobial properties of the *L. plantarum* postbiotic fraction.

## Discussion

The high prevalence of pan-drug resistant (PDR) *Salmonella enterica* isolated from both human clinical gastroenteritis cases (15% prevalence) and Egyptian retail meat samples highlights a major public health concern in the region. The identification of clinical PDR Salmonella enterica serovar Enteritidis (*S.* Enteritidis), alongside PDR *Salmonella enterica* serovar Anatum (*S.* Anatum) and *Salmonella enterica* serovar Kentucky (*S.* Kentucky) from retail meat surveillance (El-Demerdash et al., 2021), underscores the widespread dissemination of multi-drug resistant (MDR) phenotypes across the human-food interface. Molecular confirmation via *invA* gene PCR definitively validated the identity of all isolates, while comprehensive antimicrobial susceptibility testing across nine distinct antibiotic classes confirmed extensive resistance (Saad et al., 2023; Liao et al., 2026). Although these data demonstrate a shared reservoir of resistance traits across clinical and food supply chains, they do not establish direct transmission between these sources (Despotovic et al., 2023; El-Demerdash et al., 2023a, 2026; Emad et al., 2026). Instead, they emphasize the necessity of developing non-antibiotic biocontrol interventions capable of neutralizing PDR *Salmonella* strains irrespective of their point of origin.

To elucidate the biochemical basis underlying these observed biological activities, the cell-free spent medium (CFSM) of *L. plantarum* CCUG 30503 was subjected to quantitative organic acid profiling via High-Performance Liquid Chromatography (HPLC). Liquid-phase chromatography with UV detection at 210 nm provided superior thermal stability and chromatographic resolution compared to gas-phase techniques, eliminating thermal decarboxylation artifacts and permitting precise quantification of non-volatile organic acids. The postbiotic fraction revealed a rich multi-acid profile dominated by primary fermentation end-products alongside metabolic intermediates, including lactic acid (1.89 mg/mL), succinic acid (1.53 mg/mL), acetic acid (1.11 mg/mL), oxalic acid (0.47 mg/mL), glycolic acid (0.43 mg/mL), and malic acid (0.30 mg/mL).

This chemical composition underscores a foundational advantage in the transition from live probiotic organisms to postbiotics—defined here as inanimate microbial fractions and bioactive metabolites operating via targeted phenotypic and genetic attenuation (Rocchetti et al., 2024; Abdelaziz et al., 2025; El-Demerdash et al., 2025).Unlike live probiotics, which face viability limitations, potential safety risks in immunocompromised hosts, and the risk of horizontal gene transfer, postbiotics offer greater physical stability and operate via targeted phenotypic and genetic attenuation (Eraghieh Farahani et al., 2025; Pandey et al., 2025). The robust yield of these primary organic metabolites reflects the optimal nutritional architecture of De Man, Rogosa, and Sharpe (MRS) growth medium, where complex nitrogenous sources and essential trace minerals (such as manganese) drive bacterial pyruvate metabolism toward high-density organic acid biosynthesis (Janovick et al., 2025; Jona et al., 2025).

Crucially, the antimicrobial efficacy of this postbiotic fraction relies on the cooperative dynamics of this multi-acid cocktail rather than the action of a single component. While lactic acid acts as the principal permeabilizer of the outer lipopolysaccharide (LPS) layer in Gram-negative pathogens, weaker organic acids with higher pK_a_ values (such as acetic and succinic acids) remain predominantly undissociated at lower environmental pH levels, facilitating rapid passive diffusion across the hydrophobic inner membrane (Alakomi et al., 2000; Ebrahem et al., 2024).Once inside the neutral cytosol (pH≈7.5), these acids dissociate into free hydrogen ions (H^+^) and conjugate anions, collapsing the transmembrane proton motive force (Δp), inducing severe intracellular acid stress, and disrupting cellular energy metabolism (Papadimitriou et al., 2016; Hashem et al., 2024; He et al., 2025). This multi-acid synergy exerts broad-spectrum intracellular toxicity, preventing the rapid evolution of acid-tolerance responses frequently observed with single-agent organic acid exposures.

Direct morphological evidence of this membrane-disruptive activity was confirmed via Transmission Electron Microscopy (TEM). Ultrastructural examination of CFSM-treated *Salmonella* cells revealed severe morphological alterations at sub-inhibitory concentrations (SIC), including cell wall disintegration, cytoplasmic membrane detachment, and extensive leakage of intracellular contents, compared to the intact, well-defined envelope structure of untreated control cells (Elashkar et al., 2024).

Beyond growth inhibition, our findings demonstrate a significant reduction in biofilm formation, which represents a key environmental persistence mechanism for *Salmonella* across food processing surfaces. Chemical profiling via HPLC suggests that this phenotypic suppression is driven by sub-inhibitory concentrations of the multi-organic acid cocktail—dominated by lactic, succinic, and acetic acids—which act as environmental stressors that downregulate key virulence regulons.

The underlying molecular driver of this biofilm suppression is the marked transcriptomic downregulation of the master regulator *csgD*, which dropped to a fold-change of 0.002 in *S.* Anatum following treatment with *L. plantarum* CFSM:

- Structural Integrity Blockade: CsgD controls the transcription of Curli fimbriae and cellulose synthesis. By repressing *csgD*, postbiotic organic acids prevent the downstream expression of *csgA* (encoding the major subunit) and *csgB* (the nucleator protein) (Azam and Khan, 2022; Ratnikava et al., 2025). Without these extracellular matrix components, *Salmonella* cannot construct the amyloid-cellulose network necessary for surface adherence (Palika et al., 2020; Sharaf et al., 2022; El-Demerdash et al., 2023c).
- Quorum Quenching: Furthermore, the significant downregulation of *sdiA* (to 0.02) indicates a notable quorum-quenching effect (Smith et al., 2011). Organic acid-induced cytoplasmic stress impairs intercellular communication by preventing the SdiA receptor from binding *N*-acyl homoserine lactone (AHL) signaling molecules (Lazar et al., 2023; Elshobary et al., 2025), thereby disrupting population-wide coordination required for swarming and organized biofilm maturation.

The physiological impact of this acid-mediated transcriptomic downregulation is further supported by the Gentamicin Protection (Invasion) Assay using human Caco-2 intestinal epithelial cells. We observed a 3.1-log reduction in internalized *S.* Kentucky, with invasion efficiency decreasing to 0.08% compared to untreated controls. This marked reduction in invasive capacity directly correlates with the simultaneous molecular repression of the *Salmonella* Pathogenicity Island 1 (SPI-1) regulon, which encodes the Type III Secretion System (T3SS)—the primary apparatus utilized by *Salmonella* to inject effector proteins into host cells (Eade et al., 2019; Lawrence et al., 2021).

This attenuation mechanism operates sequentially across the SPI-1 cascade:

1. Master Transcriptional Regulation: Downregulation of *hilA* (to 0.188) suppresses the central genetic trigger required to initiate T3SS needle complex assembly (Manisha et al., 2024).
2. Structural Assembly Inhibition: This repression is accompanied by the downregulation of *invF* (to 0.001) and associated structural components, including *prgH* (Lv et al., 2022). Without the PrgH-PrgI-PrgK structural complex, *Salmonella* cannot properly assemble the extracellular needle filament required to penetrate host cell membranes (Romero-González et al., 2020).
3. Effector Protein Suppression: Finally, the downregulation of the effector gene *sipA* (to 0.037) reduces the actin-binding capacity necessary for host cell cytoskeletal rearrangement (McGhie et al., 2001). By suppressing SipA expression, the organic acid-rich CFSM prevents the actin polymerization and membrane ruffling required for Caco-2 host cells to engulf the pathogen (Zha et al., 2019; Mkangara et al., 2020; Mkangara, 2023).

When compared with recent postbiotic literature (Neelawala et al., 2024; Bustos and Gerez, 2026; Ranjbar et al., 2026), which primarily emphasizes general growth inhibition, our findings indicate that probiotic postbiotics act directly on the regulatory networks governing *Salmonella* virulence. By downregulating both structural apparatus components (T3SS) and key regulatory networks (*csgD, sdiA, hilA, invF, prgH, sipA*) in pan-resistant strains, probiotic-derived postbiotics present a targeted biocontrol strategy that mitigates pathogen virulence while avoiding the selective pressure associated with conventional antimicrobials.

## Conclusion

The present investigation establishes that postbiotic cell-free spent media (CFSM) derived from *Lactobacillus* species—with *Lactobacillus plantarum* CCUG 30503 identified as the superior lead candidate—exert potent antimicrobial, antibiofilm, and antivirulence activities against pan-drug resistant (PDR) *Salmonella enterica* serovars isolated from poultry and human clinical settings. Quantitative HPLC profiling attributed this bioactivity to a synergistic multi-organic acid cocktail dominated by lactic (1.89 mg/mL), succinic (1.53 mg/mL), and acetic (1.11 mg/mL) acids. At sub-inhibitory concentrations (10% v/v), the postbiotic fraction induced transcriptomic silencing of master regulators governing biofilm architecture (*csgD*), quorum sensing (*sdiA*), and SPI-1 T3SS invasion (*hilA*, *invF*, *sipA*). This molecular suppression directly correlated with severe ultrastructural membrane damage (TEM) and a drastic reduction in Caco-2 cell invasion efficiency (down to 0.08%). Collectively, these findings establish *L. plantarum* CCUG 30503 postbiotics as a potent, non-antibiotic biocontrol modality against PDR *Salmonella* across the food safety and public health continuum.

## Data Availability

The datasets presented in this study can be found in online repositories. The names of the repository/repositories and accession number(s) can be found in the article/**Supplementary Material**.
