# Supplementary material for: Disrupting Salmonella colonization and resistance: probiotic cell-free supernatants inhibit biofilm formation and invasion in meat and human isolates
Source: Front Cell Infect Microbiol. 2026 Oct 2;16:1889179. doi: 10.3389/fcimb.2026.1889179 (PMC13671930; doi:10.3389/fcimb.2026.1889179)
Supplement: Supplementary file 1 [file Supplementaryfile1.docx]

**Table S1.** Minimum Inhibitory Concentration (MIC) Distribution and Resistance Profiles of Nine Pan-Drug Resistant (PDR) Salmonella Isolates Relative to CLSI Breakpoints

| **Antimicrobial Class** | **Representative Antibiotic** | **CLSI Resistant Breakpoint (μg/mL)** | **S. Enteritidis Clinical (n=3)** | | | **S. Anatum Meat (n=3)** | | | **S. Kentucky Meat (n=3)** | | | **Statistical Metrics (All Isolates, N=9)**  **Geometric Mean (μg/mL)** | **MIC90 (μg/mL)**  **MIC90 (μg/mL)** | **Phenotypic Profile** |  |  |  |
| --- | --- | --- | --- | --- | --- | --- | --- | --- | --- | --- | --- | --- | --- | --- | --- | --- | --- |
| Aminoglycosides | Gentamicin | ≥16 | 64 | 32 | 64 | 32 | 64 | 32 | 128 | 64 | 128 | 57.02 | 128 | Resistant |  |  |  |
| Penicillins | Ampicillin | ≥32 | 128 | 128 | 128 | 128 | 128 | 128 | 128 | 128 | 128 | 128 | 128 | Resistant |  |  |  |
| β-Lactam/Inhibitor | Amoxicillin-clavulanic acid | ≥32/16 | 64/32 | 32/16 | 64/32 | 64/32 | 64/32 | 32/16 | 128/64 | 64/32 | 128/64 | 57.02/28.51 | 128/64 | Resistant |  |  |  |
| Extended-Cephalosporins | Ceftriaxone | ≥4 | 64 | 32 | 64 | 128 | 64 | 64 | 128 | 128 | 256 | 76.11 | 128 | Resistant |  |  |  |
| Carbapenems | Meropenem | ≥4 | 16 | 8 | 16 | 16 | 16 | 8 | 32 | 16 | 32 | 16.00 | 32 | Resistant |  |  |  |
| Fluoroquinolones | Ciprofloxacin | ≥1 | 8 | 4 | 8 | 16 | 8 | 16 | 32 | 16 | 32 | 11.31 | 32 | Resistant |  |  |  |
| Folate Antagonists | Trimethoprim-sulfamethoxazole | ≥4/76 | 16/304 | 16/304 | 16/304 | 16/304 | 16/304 | 16/304 | 16/304 | 16/304 | 16/304 | 16/304 | 16/304 | Resistant |  |  |  |
| Phenicols | Chloramphenicol | ≥32 | 64 | 32 | 64 | 128 | 64 | 64 | 64 | 128 | 128 | 71.84 | 128 | Resistant |  |  |  |
| Tetracyclines | Tetracycline | ≥16 | 64 | 64 | 32 | 128 | 64 | 128 | 128 | 128 | 256 | 85.34 | 128 | Resistant |  |  |  |

Testing executed in triplicate according to CLSI M100-ED33 guidelines. Geometric Means (GM) and MIC_90_ values (concentration inhibiting ≥ 90% of isolates) represent central tendency and epidemiological thresholds across all 9 panel isolates (N=9). One-way ANOVA on log_2_-transformed MIC values confirmed that S. Kentucky strains exhibited significantly higher MICs (p < 0.05) to ciprofloxacin and gentamicin than S. Enteritidis and S. Anatum.

**Table S2**. Invasion Assay Results (Internalized Bacteria)

| Salmonella Serovar | Treatment Group | Mean Log₁₀ CFU/mL | *p*-value | Invasion Efficiency (%) | Inhibition of invasion (%) |
| --- | --- | --- | --- | --- | --- |
| *S. Kentucky* | Untreated Control | 6.20 ± 0.15^a^ | < 0.0001 | 100 | 0 |
| *S. Kentucky* | L. plantarum CFSM | 3.10 ± 0.10^c^ |  | 50± 1.61 | 50± 1.61 |
| *S. Kentucky* | L. helveticus CFSM | 4.45 ± 0.12^b^ |  | 71.77± 1.94 | 28.23± 1.94 |
| *S. Enteritidis* | Untreated Control | 6.50 ± 0.11^a^ | < 0.0001 | 100 | 0 |
| *S. Enteritidis* | L. helveticus CFSM | 3.80 ± 0.09^c^ |  | 58.46± 1.38 | 41.54± 1.38 |
| *S. Enteritidis* | L. plantarum CFSM | 4.15 ± 0.13^b^ |  | 63.85± 2 | 36.15± 2 |
| *S. Anatum* | Untreated Control | 5.80 ± 0.20^a^ | < 0.0001 | 100 | 0 |
| *S. Anatum* | L. plantarum CFSM | 4.20 ± 0.14^c^ |  | 72.41± 2.4 | 27.59± 2.4 |
| *S. Anatum* | L. helveticus CFSM | 4.85 ± 0.18^b^ |  | 83.62± 3.1 | 16.38± 3.1 |

Results were expressed as mean of three independent experiments± standard error of the mean (SEM). ^a-c^ Means with different superscript letters within the same *Salmonella* serovar differ significantly at *p* < 0.05.

**Table S3.** Fold changes in the expressions of *hilA, prgH, invF, csgD, sipA,* and *sdiA* virulence genes among *Salmonella anatum* isolates after treatment with *Lactobacillus helveticus* and *Lactobacillus plantarum*.

| Gene | Control | | *Lactobacillus helveticus* | | *Lactobacillus plantarum* | | *p*-value |
| --- | --- | --- | --- | --- | --- | --- | --- |
|  | Mean | SEM | Mean | SEM | Mean | SEM |  |
| *hilA* | 1 | 0 | 0.3635 | 0.0059 | 0.1882 | 0.0064 | < 0.0001 |
| *prgH* | 1 | 0 | 0.1233 | 0.0058 | 0.7792 | 0.0054 | < 0.0001 |
| *invF* | 1 | 0 | 0.1134 | 0.0055 | 0.3392 | 0.0046 | < 0.0001 |
| *csgD* | 1 | 0 | 0.0548 | 0.0049 | 0.0023 | 0.0009 | < 0.0001 |
| *sipA* | 1 | 0 | 0.0791 | 0.004 | 0.0559 | 0.0046 | < 0.0001 |
| *sdiA* | 1 | 0 | 0.0396 | 0.0006 | 0.0612 | 0.0044 | < 0.0001 |

SEM: standard error of the mean.

**Table S4.** Fold changes in the expressions of *hilA, prgH, invF, csgD, sipA,* and *sdiA* virulence genes among *Salmonella enteritidis* isolates after treatment with *Lactobacillus helveticus* and *Lactobacillus plantarum*.

| Gene | Control | | *Lactobacillus helveticus* | | *Lactobacillus plantarum* | | *p*-value |
| --- | --- | --- | --- | --- | --- | --- | --- |
|  | Mean | SEM | Mean | SEM | Mean | SEM |  |
| *hilA* | 1 | 0 | 0.727 | 0.0087 | 0.4033 | 0.009 | < 0.0001 |
| *prgH* | 1 | 0 | 0.7526 | 0.0088 | 0.0124 | 0.0007 | < 0.0001 |
| *invF* | 1 | 0 | 0.9526 | 0.0089 | 0.5905 | 0.0067 | < 0.0001 |
| *csgD* | 1 | 0 | 0.1582 | 0.009 | 0.0647 | 0.01 | < 0.0001 |
| *sipA* | 1 | 0 | 0.0451 | 0.009 | 0.0587 | 0.008 | < 0.0001 |
| *sdiA* | 1 | 0 | 0.0201 | 0.007 | 0.0954 | 0.007 | < 0.0001 |

SEM: standard error of the mean.

**Table S5.** Fold changes in the expressions of *hilA, prgH, invF, csgD, sipA,* and *sdiA* virulence genes among *Salmonella kentucky* isolates after treatment with *Lactobacillus helveticus* and *Lactobacillus plantarum*.

| Gene | Control | | *Lactobacillus helveticus* | | *Lactobacillus plantarum* | | *p*-value |
| --- | --- | --- | --- | --- | --- | --- | --- |
|  | Mean | SEM | Mean | SEM | Mean | SEM |  |
| *hilA* | 1 | 0 | 0.0981 | 0.013 | 0.0718 | 0.013 | < 0.0001 |
| *prgH* | 1 | 0 | 0.0374 | 0.005 | 0.0884 | 0.004 | < 0.0001 |
| *invF* | 1 | 0 | 0.7371 | 0.014 | 0.0484 | 0.008 | < 0.0001 |
| *csgD* | 1 | 0 | 0.1856 | 0.011 | 0.0013 | 0.0001 | < 0.0001 |
| *sipA* | 1 | 0 | 0.2382 | 0.017 | 0.5664 | 0.006 | < 0.0001 |
| *sdiA* | 1 | 0 | 0.6643 | 0.015 | 0.5664 | 0.008 | < 0.0001 |

SEM: standard error of the mean.

**Figure S1.** Agarose gel electrophoresis (1.5%) showing PCR amplification of the genus-specific *invA* gene (~284 bp) for the molecular confirmation of pan-drug resistant *Salmonella enterica* isolates. **Lane L:** 100 bp DNA marker ladder. **Lane –C:** Negative control (nuclease-free water). **Lane +C:** Positive control (*Salmonella enterica* ATCC 14028 reference strain). **Lanes 1–9:** Pan-drug resistant (PDR) *Salmonella* isolates displaying the characteristic ~284 bp *invA* target product.
